# Supplementary material for: Improved assembly DC circuit breaker based on resonant current injection
Source: PLoS One. 2024 Jul 17;19(7):e0304979. doi: 10.1371/journal.pone.0304979 (PMC11253974; doi:10.1371/journal.pone.0304979)
Supplement: Attachment — Submitted filename: Reviewr comments_PONE.pdf [file pone.0304979.s001.pdf]

1. Provide specific parameter design specifications. This paper mentions CI-MPACB can cut off DC fault lines, but it does not provide specific details about the parameter design.
2. Resonant current injection is primarily designed for AC circuit breakers, DC circuits have a continuous and unidirectional flow of current, making it challenging to achieve the same resonant conditions. So how to achieve the same resonant conditions?
3. How Transient and Switching Stress are managed? In DC circuit breakers, resonant conditions may lead to high transient voltages and switching stresses. These transients can pose challenges in terms of insulation coordination and may require additional protective measures to prevent insulation breakdown and damage to the breaker components.
4. Achieving resonant current injection in DC circuit breakers often requires complex control algorithms and sophisticated electronic components. This increases the risk of malfunctions. So, what are the measures taken to rectify these malfunctions.
5. DC systems vary widely in terms of voltage levels, current ratings, and applications. Achieving a universal resonant current injection solution that is compatible with all DC systems is challenging, so how you will achieve this?
6. The additional complexity introduced by resonant current injection in DC circuit breakers may lead to increased maintenance requirements. So it may increase the cost. So, what is the remedy for this?
